# Proceedings of the American Academy of Dental Surgery

**Published:** 1875-12

**Authors:** 


AMERICAN JOURNAL

OF

DENTAL SCIENCE.

Vol. IX. THIRD SERIES—DECEMBER, 1875. No. 8.

### ARTICLE I.

### *The American Academy of Dental Surgery.*

Special meetings of October 19 and 20th, 1875.—The Papers Read—Addresses—Discussions.

(*Reported for the American Journal of Dental Science.*)

The American Academy of Dental Surgery held special meetings, in the lecture room of the College of Physicians and Surgeons, New York, on Tuesday and Wednesday, October 19th and 20th, 1875. The regular meeting also took place on the evening of the 19th. There was a morning and an afternoon session on each day. Papers were read, addresses delivered, and discussions held upon questions of special importance to the profession. Mayor Wickham was to have been present to deliver the opening address, but the pressure of official business forced him to be absent.

Letters were also read from a number of distinguished dentists of this country and Europe, expressing regret that unavoidable circumstances prevented their presence.

*The Proceedings.*—FIRST DAY.

At the opening session, the meeting was called to order by Dr. Geo. A. Perine, President of the Academy, and opened with prayer by the Rev. Hugh Miller Thompson, D. D., Rector of Christ Church, New York. Official business preventing the Mayor from attending, according to arrangement, he sent the following letter of regret:

MAYOR'S OFFICE, CITY OF NEW YORK.
October 19th, 1875.

DR. GEO. A. PERINE,
*President Am. Academy Dental Surgery.*

MY DEAR SIR:

Unexpected official business will prevent my being with you this A. M., as was my intention. With best wishes for the success and prosperity of the Academy,

I am your obd't servant,

W. H. WICKHAM, *Mayor.*

---

Dr. Geo. H. Perine delivered the address of GREETING. He said:—

*Gentlemen, Fellows of the American Academy of Dental Surgery and Invited Guests:*

In rising to address you on this occasion, I find myself face to face with a double duty. To you, Fellows of the Academy, I have to offer my congratulations, upon the advent of this incident in our organic life.

To you gentlemen, who have so kindly responded by your presence to our request that you would unite with us on this occasion, I have to tender the cordial thanks of the body over which I have the honor to preside.

Gentlemen; You are welcome, WELL-COME in a double sense. For while I am glad to recognize among you so many familiar faces of members of my own profession, it is with feelings of special satisfaction that I observe also representatives of the kindred profession,—medicine. Gen.

tlemen it is our design—God willing, to so order it in our advance as a scientific, corporate body, that those sentiments of professional cordiality which should exist between our respective guilds, shall not be smothered, but shall—at least in so far as we can be instrumental in forwarding so desirable a conclusion—become among the dearest aims and objects of our fraternity. And, gentlemen, I am glad to recognize another element in this gathering, which seems to me to betoken that fruition of our hopes which we have most at heart. I allude to the presence among us, to-day, of those younger branches of the professional tree, upon whose present and whose future must rest the advancement of our two professions hereafter; for although I stand before you as it were the representative of calm, sedate and solemn age, and from my hoary pinnacle of years must look with chastened and dispassionate gaze upon these young and daring souls; yet, gentlemen, it is with the sentiment and the full belief that it is upon them, and not upon us, must fall the burden of that progress towards which we are all striving.

And, gentlemen, I am entitled to declare that it is a main and leading object of "The American Academy of Dental Surgery" to recognize and approve and encourage the efforts and the success of our young men. For gentlemen, it is through these young men that the aim and hope to which I have already alluded can alone be made successful. Through them I hope to see such a bond of union cemented between the Medical and Dental professions that they shall become as it were one in fact, as they are now in theory and practice; and that the time shall be near at hand when a Professor of Dental Surgery in every Medical College, and stdents in the art under their tuition, shall give promise to the world of perfection in the two sciences.

Gentlemen, we are fast approaching an era in our national existence, and as we draw near to the solemn celebration of our Centennial anniversary, I think I am justified in the assertion that in no other branch of science or art can we display such marked progress and success as in the science of Dentistry. And not only have we builded here this wondrous edifice of scientific attainment, but, gentlemen, I venture to say, that among all that vast gathering of the inhabitants of lands beyond the sea, who will assemble in Philadelphia next summer, there shall be not one who will not have witnessed in his own city the wonderful and adventurous excellence of the American Dentist.

And this excellence, purely American as it is in all its peculiarities, is not due alone to individual effort. It is due in no minor part to the wide extension, the laborious energy and the far-sighted originality of our system of organized association. Through the attrition of many minds it is that we have accomplished so much in the past; and if I may venture, as the representative of the youngest of these organizations, to compliment those which have preceeded as, I will only say that our ambition is to take up the good wor where these have left it, and to forward it with something of that earnestness and determination which have ever characterized their efforts.

Gentlemen, in closing my remarks, permit me again to congratulate "The American Academy of Dental Surgery" upon this handsome response to our invitations on this occasion. In this connection I would have desired especially to express the gratification with which we could have welcomed the presence of the Mayor of our city. But it is seldom that high civic functionaries can borrow the time from their arduous avocations to represent their constituents in scientific gatherings, and our Mayor in this instance, has been compelled to make no exception to the general rule.

Gentlemen, it is given to none of us to predict the future; but I think I may say, in view of the success of this meeting, that however the science of Dentistry has progressed in the hands of American Dentists in the past, we may look forward with bright hopes to a future of still broader conception, and of still grander execution.

Prof. J. M. Carnochan, M. D., New York, then made some extemporaneous remarks upon dentistry, past and present. He said:

The subject of Dentistry is one of great importance. The sacred ministers of religion in former times were supposed to minister to the physical wants of the tribes amongst which they were placed; often a time the surgical art became more or less linked with the mechanism of the barber; in the course of time it assumed a different phase; it now probably stands at the head of all those branches of science which are devoted to the benefit of the human race. Surgery has become divided into a number of departments, and amongst these is dentistry. The art of dentistry now embraces not only the mechanical use of the instruments applied to the teeth, but also a knowledge on the part of the operator of all the vital functions of the system. The mere mechanical dentist at the present day, would be considered a very unfit member of the dental profession. To dentistry surgery owes one of the greatest boons that science has given to humanity. To Horace Wells, the dentist of Hartford, is due the discovery of Anæsthesia. Dental surgery has done much for general surgery. In the deformity known as hare-lip, for instance, the operation of staphyloraphy is now seldom resorted to, because of the great improvement upon that operation invented by Sterns, the dentist. The dental art is so associated with the surgery of the jaw, that it is difficult to separate the one from the other. The disease known as *tic douloreux* certainly comes under the head of the dental art, and medical men expect to hear of improvements made by dentists in the treatment of this fearful disease.

The dental art has now assumed so important a position, that it must not be excluded from the domain of surgery. It is no longer a mechanical art—it is based upon the science of vitality; and it must and will again become a part of the general profession. There is no seat in medicine but what has been of some advantage to the general art of healing. Dentistry must cease to be an abstraction from this general art. [Applause.]

At the conclusion of Dr. Carnochan's address, the letters already mentioned were read, and then an adjournment was had to 1-30 P. M.

*Afternoon Session.*

The afternoon session was opened with a paper on "Reflex Nervous Disorders from Dental Irritation." By Alfred L. Carroll, M. D., New York.

This was a very able paper, and was listened to with a great deal of interest. He showed that the pathological territory upon which dental surgery and pure medicine met was wider than was commonly imagined, and he urged upon young physicians that in the words of a most observant medical writer, (Tanner,) the importance of investigating the state of the mouth in all cases of neuralgia about the face and head, headache, deafness, amaurosia, facial paralysis, epilepsy, abscess of the antrum, and hypochrondriasis, cannot be too strongly insisted upon; while to the young dental surgeon he commended the propriety of recognizing the nervous system as an appendage to the teeth, and bearing in mind the reflex disorders which, in certain temperaments, his very skill too specially applied, may possibly perpetuate.

The paper was so logically constructed—the reasoning so close, that any attempt to give an abstract of it does injustice to the writer.

A discussion upon the subject of the paper followed, and then Prof. Wm. A. Hammond, M. D., medical department Uuiversity of New York, was introduced. His subject was

"THE RELATIONS OF DENTAL SCIENCE TO MEDICINE."

He said: Mr. President and Gentlemen of the American Academy of Dental Surgery. The cordial sympathy of the medical profession is with you in the effort in which you are engaged. Medical science embraces within its fold all those who are devoted to the alleviation of the sufferings of mankind; and if we look to the ills which will follow derangement of the teeth, and to the good which is accomplished by their cure, we are bound to accord to dentists a very high position amongst those engaged in similar labors. At one time there was no difference between the dentist and the surgeon. This was undoubtedly disadvantageous to their patients. If there is anything which tends to the advancement of any one department of medical science, it is the separation of that department from the mass by those who take a special interest in it. Prior to the separation of dentistry from surgery proper, not much progress was made in any scientific department. Here is a volume some three hundred years old, written by Ambroise Paré. Let us see how they performed operations in his day.

[The Doctor proceeded to read extracts from the volume, showing the state of dental knowledge three hundred years ago. From the stand point of modern science, many of the old surgeon's directions, (such for instance, as the taking of oil of vitriol into the mouth for toothache,) seemed ludicrous indeed; while his quaint remarks produced much laughter.]

Gentlemen, I think dentists should not separate themselves from surgery proper. We may differentiate too much. In striving to enlarge the bounds of science, have we not, to some extent, wandered from the pathway through it? That there is no actual difference between medicine and dentistry is very evident. The question arises is it wise to have dental colleges? Would it not be better to have chairs of dentistry in medical colleges, where the student would not only become proficient in dentistry, but obtain a full knowledge of medicine—without which no man can become an accomplished dentist. [Applause.]

That brings me to the consideration of the relations which naturally exist between these two departments of science. We have one common ground between us—the fifth pair of nerves. All surgeons work upon it more or less—all dentists work upon it entirely. Is the dentist who is unacquainted with anatomy, physiology, pathology and therapeutics, competent to point out to his patients what they are to do? You will agree with me that he is not.

The relations of medicine to dentistry are not only very manifest as regards the prominent diseases which we have to treat, but likewise as regards the contributions which have been made to the common science of healing. Considering the limited field that the dentists occupy, they have contributed more to anatomy, histology and therapeutics than any other class of medical men. We leave the histology of the teeth entirely to the dentists. Dentists have been large contributors to our stock of knowledge relating to diseases of the teeth, jaw, palate and tongue.

The differentiation I spoke of is a bad move. At the present day no such differentiation should be made. It is not carried in Europe to the same extent that it is here. A surgeon who prefers to practice one branch of surgery is just as much a surgeon as if he practiced general surgery. A surgeon-dentist is as much a medical man as a general practitioner.

*Discussion.*

Dr. William H. Atkinson, New York, said: Gentlemen, when it comes to differentiation between the two professions as to whether I shall call myself a medical man or a dentist, I will stand with the dentists. But lest the medical gentlemen present should not apprehend the warm affection I have to all interested in medicine, I would like to explain. In an address I once delivered, I said that I for one would require candidates for dentistry to be first A. B., next M. D.'s, and then we would give them dental skill, and finally bring them to a proper institution to be trained in dental surgery. We cannot be perfect, but we can advance towards perfection.

Dentistry has no reason to blush in the face of medicine. The army records show that the surgical operations of the dentists showed those of the best surgeons. A pupil of mine, who has passed "behind the curtain," once performed in the army, the operation of amputation of the hip joint, and so dexterously was it done, that several of the surgeons came up and complimented him upon it. Said one, "That is not your maiden operation by any means!" "You have made that operation many times before; for there is no man living who could have made it without having gone over it again and again upon the living body." He had never performed the operation upon the living body before.

Dr. Howard, New York, said Dr. Atkinson's remarks come home to me in regard to the delicate skill which the dentist is called upon to exercise. Prof. Darling, of the medical department University of New York, once told me that those students who had experience in the dental art made the best surgeons that they graduated from their college. The delicate touch of the dentist caused by experience, and we recognize this touch in every operation we perform.

Dr. Miles, Philadelphia, said the touch spoken of is a most radical accomplishment. Some men are unfitted by nature to acquire it. How often do we hear the remark, "He is so rough, I will never go back to that dentist!" The very touch of the man is repugnant, and although he may have done his work well, he is disparaged because of his awkardness.

Dr. John M. Riggs, Hartford, Conn., said the surgeon must be born a surgeon. A gross-minded man never can make a good surgeon. The motto of the poet applies here —*poeta nascitur non fit.* This is eminently true of the dentist. At the present day, we need the nicest manipulation. The dentist, as well as the surgeon, must have his eyesight at the ends of his fingers.

The next paper read was entitled, "The Tooth Brush as a Prophylactic." By Abram Robertson, M. D., F. A. A. D. S., Newport, R. I. The paper was read by Dr. Merrill, for the author, who is blind. A discussion upon one or two points raised in the paper followed, and then the meeting adjourned, to reassemble at the house of the President, 50 W. 35th St., at 7-30 P. M.

TO BE CONTINUED.

